# Role of PET/CT and Therapy Management of Pancreatic Neuroendocrine Tumors

**DOI:** 10.3390/diagnostics10121059

**Published:** 2020-12-07

**Authors:** Diletta Calabrò, Giulia Argalia, Valentina Ambrosini

**Affiliations:** 1 Department of Nuclear Medicine, IRCCS Azienda Ospedaliera-Universitaria di Bologna, 40138 Bologna, Italy; giulia.argalia@studio.unibo.it (G.A.); valentina.ambrosini@unibo.it (V.A.); 2 Department of Nuclear Medicine, DIMES University of Bologna, 40138 Bologna, Italy

**Keywords:** panNET, pancreatic, neuroendocrine, PET/CT, [^68^Ga]Ga-DOTA, [^18^F]FDG, PRRT, [^18^F]FDOPA

## Abstract

Pancreatic neuroendocrine neoplasms (panNENs) are heterogeneous neoplasms with neuroendocrine differentiation that show peculiar clinical and histomorphological features, with variable prognosis. In recent years, advances in knowledge regarding the pathophysiology and heterogeneous clinical presentation, as well as the availability of different diagnostic procedures for panNEN diagnosis and novel therapeutic options for patient clinical management, has led to the recognition of the need for an active multidisciplinary discussion for optimal patient care. Molecular imaging with positron emission tomography/computed tomography (PET/CT) has become indispensable for the management of panNENs. Several PET radiopharmaceuticals can be used to characterize either panNEN receptor expression or metabolism. The aim of this review is to offer an overview of all the currently used radiopharmaceuticals and of the new upcoming tracers for pancreatic neuroendocrine tumors (panNETs), and their clinical impact on therapy management. [^68^Ga]Ga-DOTA-peptide PET/CT (SSA-PET/CT) has high sensitivity, specificity, and accuracy and is recommended for the staging and restaging of any non-insulinoma well-differentiated panNEN cases to carry out detection of unknown primary tumor sites or early relapse and for evaluation of in vivo somatostatin receptors expression (SRE) to select patient candidates for peptide receptor radiometabolic treatment (PRRT) with ^90^Y or ^177^Lu and/or cold analogs. SSA-PET/CT also has a strong impact on clinical management, leading to a change in treatment in approximately a third of the cases. Its role for treatment response assessment is still under debate due to the lack of standardized criteria, even though some semiquantitative parameters seem to be able to predict response. [^18^F]FDG PET/CT generally shows low sensitivity in small growing and well-differentiated neuroendocrine tumors (NET; G1 and G2), while it is of utmost importance in the evaluation and management of high-grade NENs and also provides important prognostic information. When positive, [^18^F]FDG PET/CT impacts therapeutical management, indicating the need for a more aggressive treatment regime. Although FDG positivity does not exclude the patient from PRRT, several studies have demonstrated that it is certainly useful to predict response, even in this setting. The role of [^18^F]FDOPA for the study of panNET is limited by physiological uptake in the pancreas and is therefore not recommended. Moreover, it provides no information on SRE that has crucial clinical management relevance. Early acquisition of the abdomen and premedication with carbidopa may be useful to increase the accuracy, but further studies are needed to clarify its utility. GLP-1R agonists, such as exendin-4, are particularly useful for benign insulinoma detection, but their accuracy decreases in the case of malignant insulinomas. Being a whole-body imaging technique, exendin-PET/CT gives important preoperative information on tumor size and localization, which is fundamental for surgical planning as resection (enucleation of the lesion or partial pancreatic resection) is the only curative treatment. New upcoming tracers are under study, such as promising SSTR antagonists, which show a favorable biodistribution and higher tumor-to-background ratio that increases tumor detection, especially in the liver. [^68^Ga]pentixafor, an in vivo marker of CXCR4 expression associated with the behavior of more aggressive tumors, seems to only play a limited role in detecting well-differentiated NET since there is an inverse expression of SSTR2 and CXCR4 in G1 to G3 NETs with an elevation in CXCR4 and a decrease in SSTR2 expression with increasing grade. Other tracers, such as [^68^Ga]Ga-PSMA, [^68^Ga]Ga-DATA-TOC, [^18^F]SiTATE, and [^18^F]AlF-OC, are also under investigation.

## 1. Introduction

Pancreatic neuroendocrine neoplasms (panNENs) are heterogeneous neoplasms with neuroendocrine differentiation that show peculiar clinical and histomorphological features, with variable prognosis. Up to 10% of panNENs develop in patients with syndromes such as multiple endocrine neoplasia type 1 (MEN1), von Hippel–Lindau disease (VHLD), tuberous sclerosis complex, neurofibromatosis type 1, and glucagon cell adenomatosis. Most panNENs are non-functioning and are usually diagnosed following the occurrence of mass-related symptoms. On the contrary, a minority of panNENs clinically present early due to symptoms secondary to increased hormone production [1]. Clinical presentation and outcome are strongly influenced by tumor differentiation. The 2010 WHO classification categorized panNENs as grade 1 and grade 2 pancreatic neuroendocrine tumors (panNETs), and grade 3 pancreatic neuroendocrine carcinomas (panNECs), based on the Ki-67 index or the mitotic index (G1: Ki-67 < 3%, G2: Ki-67 3–20%, G3: Ki-67 > 30%). The WHO panNEN grading system was later revised in 2017 to include panNETs (G1: Ki-67 < 3%, G2: Ki-67 3–20%, G3: Ki-67 > 30%) and PanNECs (Ki-67 < 20%, including small-cell and large-cell types). These are genetically distinct entities with different clinical presentations, prognoses, and therapeutic options [2,3]. The most recent WHO 2019 classification introduced a new distinction for neuroendocrine neoplasms with Ki-67 > 20% in two groups, well-differentiated panNET grade 3 and poorly differentiated small and large-cell type panNECs, based on the morphologic characteristics [4,5]. panNENs account for 5% of all pancreatic tumors, but their incidence is on the rise, most likely due to a combination of increased clinical awareness, more accurate and rapidly evolving diagnostic procedures, and increased incidental findings [6]. In recent years, advances in knowledge of the pathophysiology, heterogeneous clinical presentation, availability of different diagnostic procedures for panNENs diagnosis, and novel therapeutic options for patients’ clinical management has led to a recognition of the need for an active multidisciplinary discussion involving different specialists (oncologists, nuclear medicine physicians, surgeons, radiologists, pathologists, and radiotherapists) for optimal patient care. In fact, the current ESMO and ENETS guidelines [4,5] encourage a multidisciplinary approach, and there is growing literature supporting the increased quality of NET care related to multidisciplinary team activity [6,7,8,9,10,11,12].

Molecular imaging with positron emission tomography/computed tomography (PET/CT) has become indispensable for panNEN management [3]. Several PET radiopharmaceuticals can be used to characterize either panNEN receptor expression or metabolism. The purpose of this review is to offer an overview of all currently used radiopharmaceuticals and of new upcoming tracers for panNENs and their clinical impact on therapy management, especially focusing on panNETs.

## 2. [^68^Ga]Ga-DOTA-Peptides PET/CT

**Figure 1:**
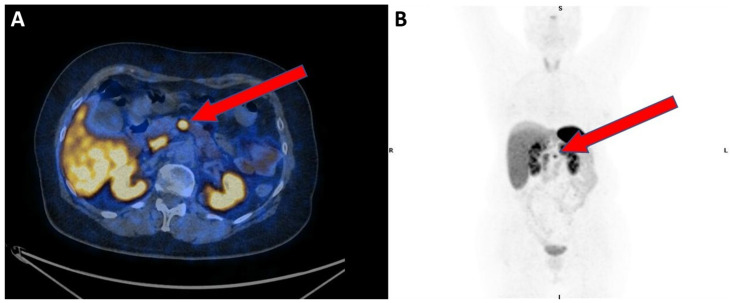
Presurgical [^68^Ga]Ga-DOTANOC PET/CT transaxial (**A**) and MIP (**B**) show focal and intense uptake in the primary pancreatic lesion (red arrows).

Somatostatin receptor expression (SRE) can be demonstrated in vivo by means of somatostatin receptor scintigraphy (SRS), somatostatin receptor single-photon emission computed tomography (SPECT/CT), or 68Ga-labelled somatostatin analogs (SSA-PET). While the “old-fashioned” somatostatin receptor scintigraphy (SRS), even if acquired with SPECT/CT, may still be used if PET/CT is not available, numerous studies have demonstrated the superior sensitivity and specificity of SSA-PET for localizing panNETs. In various studies on panNETs, PET/CT sensitivity varied from 86% to 100%, and specificity ranged from 79% to 100% [13,14,15,16,17,18,19,20] for all panNETs, except insulinomas, in which case the sensitivity was only 25% [21]. As a consequence, current 2016 ENETS guidelines for the management of panNETs [22], 2017 ENETS guidelines for radiological, nuclear medicine, and hybrid imaging [23] of neuroendocrine tumors and 2017 EANM [24] guidelines consider SSA-PET/CT, if available, as the first-line diagnostic procedure for staging or restaging any non-insulinoma panNET case for detection of the unknown primary tumor site or early relapse, and for evaluation of in vivo SRE to select patients candidate for peptide receptor radiometabolic treatment (PRRT) with ^90^Y (Yttrium-90) or ^177^Lu (Lutetium-177) and/or cold somatostatin analogs [1,2]. Three different radiopharmaceuticals are clinically available: [68Ga]Ga-DOTATATE (DOTA,Tyr(3)-octreotate), [^68^Ga]Ga-DOTANOC (DOTA,1-Nal(3)-octreotide), and [^68^Ga]Ga-DOTATOC (DOTA, D-Phe1, Tyr (3)-octreotide), showing different affinity for somatostatin receptor (SSTR) subtypes. In particular, [^68^Ga]Ga-DOTATATE shows the highest affinity for SSTR2, DOTATOC has a high affinity for SSTR2 but also for SSTR5, while DOTANOC shows a high affinity for SSTR2 but also for SSTR3 and SSTR5 [25]. Currently, no clinically relevant differences have been reported among the different tracers, although semiquantitative parameters are not directly comparable. Although SSA-PET/CT is a highly sensitive and specific technique for neuroendocrine tumor (NET) detection, various physiologic and pathologic processes may show SSTR expression. False positives include physiological uptake in the pancreatic uncinate process, accessory spleens (including intra-pancreatic accessory spleen), splenules, infectious/inflammatory findings (due to increased SSTR expression on activated lymphocytes), and non-neuroendocrine tumors (including breast carcinoma, melanoma, lymphoma, prostate carcinoma, non-small cell lung cancer, head and neck cancer, sarcoma, renal cell carcinoma, differentiated thyroid carcinoma, and astrocytoma) [24]. False negatives are observed in cases of lesions under PET/CT spatial resolution, in undifferentiated lesions, or at sites of physiological biodistribution [26]. Pancreatic uncinate process uptake is visualized in up to one-third of patients and may present with either a diffuse or a focal pattern. While in the case of a diffuse pattern, the interpretation is relatively straightforward, the interpretation of a focal uptake may be more challenging, especially for inexperienced readers. EANM guidelines recommend to interpret uncinated process uptake as physiologic in the absence of a corresponding morphological abnormality on the corresponding CT images [24]. Even if rare, intrapancreatic accessory spleens can also mimic a panNET [27]. In equivocal cases, [99mTc]Tc heat-damaged red blood cell scintigraphy [28] or [99mTc]Tc-colloid SPECT/CT [29] may be used for the differential diagnosis. A recent systematic review [30] outlined the utility of SSA-PET/CT for the staging of panNETs, demonstrating a high detection rate and diagnostic performance (Figure 1).

In this paper, 38 studies were selected for qualitative analysis, and 18 papers were included in the meta-analysis. The number of panNET patients ranged from 10 to 142 across the included studies, with a total of 1143 subjects. The patient-based analysis, pooled sensitivity, and specificity for the assessment of primary panNET were 79.6% (95% confidence interval (95% CI): 71–87%) and 95% (95% CI: 75–100%), respectively [30]. Pooled detection rate for the primary lesion was 81% (95% CI: 65–90%) and 92% (95% CI: 80–97%) for patient-based and lesion-based analysis, respectively [30]. SSA-PET/CT proved to be more sensitive than conventional imaging for the detection of distant metastasis, in particular at bone level: on a patient basis analysis, PET/CT showed higher sensitivity (100% vs. 80%), specificity (100% vs. 98%), positive predictive value (100% vs. 92%), and negative predictive value (100% vs. 95%) for the detection of bone metastasis [31]. Sharma et al. evaluated the accuracy of [^68^Ga]Ga-DOTANOC PET/CT for staging and restaging in 178 scans of panNET patients. The overall sensitivity, specificity, and accuracy of [^68^Ga]Ga-DOTANOC PET/CT were 85.7%, 79.1%, and 84.8%. The corresponding values were 73%, 50%, and 70.4% for diagnosis/staging groups and 98.6%, 100%, and 98.8% for restaging groups. The accuracy of PET/CT was significantly higher in the restaging group compared to the diagnosis/staging group (98.8% vs. 70.4%; *p* < 0.0001). However, when the analysis was carried out excluding insulinoma patients (a condition well known to affect SSA-PET/CT diagnostic accuracy) from the diagnosis/staging group, no differences between the groups were reported (98.8% vs. 94.8%; *p* = 0.349) [13]. Several papers reported the high accuracy of SSA-PET/CT for the detection of unknown primaries in patients with confirmed secondary NET lesions [32,33,34]. Therefore, for the majority of carcinoma of unknown primary (CUP) patients presenting with advanced metastatic disease at the time of diagnosis, timely identification of occult primary tumors was essential for clinical decision-making [35]. Specifically, regarding panNETs, resection of the primary tumor in the setting of diffuse disease, especially when unresectable liver disease is present, has been suggested to be beneficial for long-term patient survival but requires strict selection criteria and a multidisciplinary approach [36]. Haug et al. suggested the substantial role of [^68^Ga]Ga-DOTATATE PET/CT in the follow-up of 33 NET patients, including nine panNETs, after curative resection. [^68^Ga]Ga-DOTATATE PET/CT helped exclude recurrent NET, with 90% and 82% sensitivity and 81% and 90% specificity for PPV and NPV, respectively [37]. Several studies also demonstrated a potential prognostic role of [^68^Ga]Ga-DOTA-peptides in the field of panNETs. Several prognostic factors were identified, including maximal standardized uptake value of the most intense focus (SUVmax) and total functional tumor volume (TFTV) [38,39]. Ambrosini et al. examined 43 patients with panNETs and found that one of the major risk factors for progression included an SUVmax of no more than 37.8 (hazard ratio 3.09; *p* = 0.003) [38]. In the study of Ohnona et al., multivariate analysis determined that TFTV greater than 13.8 cm3 was the only criterion considered a significant risk factor for tumor progression (hazard ratio 2.9; *p* = 0.0003) in 50 patients with panNETs. A recent study suggested that, since SSA-PET/CT imaging and lanreotide share the same SSTR targets, [^68^Ga]Ga-DOTA-TOC PET/CT may have prognostic implications in association with the efficacy of lanreotide for well-differentiated unresectable or metastatic gastroenteropancreatic (GEP)-NETs. In particular, tumor to liver ratio (TLR), calculated as SUVmax divided by SUVmean of the liver, proved to be the only independent prognostic factor for patients who received lanreotide therapy [40]. 

The clinical impact of SSA-PET in panNETs is well established as a consequence of the receptor-based mechanisms of radiopharmaceutical uptake. In fact, it can both serve to detect the disease extent (often changing the disease stage, especially for the detection of unsuspected secondary lesions, e.g., in bone or liver) or to select the patients for treatment with cold or hot (PRRT) somatostatin analogs. Several studies demonstrated that SSA-PET/CT findings might lead to a change in the clinical management of GEP-NETs. Although NEN may arise from different GEP primary sites, the pancreas is one of the most frequent locations. Ambrosini et al. conducted a study on 90 patients with NETs, including 30 cases with panNETs. [^68^Ga]Ga-DOTANOC PET/CT affected either stage or therapy modification in 50 of the 90 patients (55.5%). In particular, the impact of [^68^Ga]Ga-DOTANOC PET/CT was particularly evident in cases in which PET/CT and conventional imaging were discordant. Discordant [^68^Ga]Ga-DOTANOC PET/CT and conventional imaging findings were observed in 42 of the 90 patients. PET resulted in a modification of either stage identification or therapy in 32 patients (76.2%). Moreover, PET/CT was also useful in preventing inefficient targeted therapy in two patients who lacked SSTR expression [16]. Similar results were obtained by Skoura et al. [41] in a population of 728 NET patients studied to assess the impact of [^68^Ga]Ga-DOTATATE PET/CT on treatment decision and survival. In the subgroup of 142/728 with panNET, PET/CT impacted the management in almost 30% of the cases. The most frequent change was a switch from surgery to chemotherapy or PRRT. Recently, in 101 GEP-NET patients (24/101 with panNETs), SSA-PET/CT findings influenced the management in one-third of the cases. In particular, in the surgery subgroup, the impact on management was reported in half of the patients [42]. Similar results were obtained in another study conducted on 114 patients with pancreatic and small bowel neuroendocrine tumors: SSA-PET/CT led to a major change in the management in almost half of the patients [43]. The use of [^68^Ga]Ga-DOTATATE PET/CT with contrast enhancement (CECT) was associated with an increased diagnostic accuracy for the detection of extra-hepatic NET secondary lesions compared to stand-alone CECT. Especially for bone and nodal lesions, sensitivity and specificity could be significantly increased, resulting in an upstaging of the disease and/or change in clinical management in >25% of patients [44]. The demonstration of high SSTR expression by [^68^Ga]Ga-DOTA-peptides PET/CT is also mandatory before PRRT in both functioning and non-functioning-panNETs [22,45]. For many years, data about PRRT efficacy and safety has been derived from only a few early-phase trials or retrospective studies, often with small patient cohorts. Although encouraging, the lack of standardized protocols for PRRT administration (in terms of number of cycles, total dose, dose per cycle, time interval between cycles, type of radiopharmaceutical, etc.) has limited the recognition of its efficacy and delayed its registration among available treatment options. The first phase 3, randomized, double-blind, multicenter trial (NETTER-1) on the use of PRRT was published in 2017 [46] and demonstrated markedly longer progression-free survival (PFS) in the [^177^Lu]Lu-DOTATATE-treated arm (in combination with standard-dose octreotide) as compared to the off label use of high-dose octreotide in patients with advanced midgut NETs. Following the publication of this study in 2017, these promising results led the Food and Drug Administration (FDA), European Medicines Agency (EMA), and the National Institute for Health and Care Excellence to approve the use of [^177^Lu]Lu-DOTATATE in SSTR-positive well-differentiated GEP-NETs, at a recommended fixed dosage of 7.4 GBq (200 mCi) every 8 weeks, for a total of four cycles. Among the advantages of PRRT, it is mandatory to report the relatively low prevalence of severe adverse events. Although PRRT represents the latest and most promising treatment option for patients with well-differentiated NETs, including in the pancreas, many issues are still to be elucidated, particularly regarding the most appropriate timing of PRRT as compared to other treatment options, combination therapies, late adverse effects, response assessment criteria, and dose personalization (in particular, some papers also document good efficacy in cases treated with lower doses, offering this treatment option to patients otherwise excluded due to renal impairment) [47,48]. A recent review collected results from eight different studies on the efficacy of PRRT in panNET patients: the pooled analysis demonstrated that the median PFS ranged from 20 to 39 months and the median overall survival (OS) ranged from 37 to 79 months. In contrast to the clinical evidence of different prognosis depending on the primary tumor site, the authors reported no significant differences in PFS or OS when comparing panNETs to other primary NET sites, even though the studied cases were quite heterogeneous for both previous lines of therapy as well as whether patients had progressive disease when treated [49]. Published data also addressed the issue of the potential use of PPRT, both in the neoadjuvant and the adjuvant setting for panNET treatment. In the study conducted by Vliet et al. [50], nine of the 29 patients (31%) with a borderline or unresectable pancreatic primary tumor (due to vascular involvement) before PRRT were successfully surgically treated after PRRT. In contrast, Bertani et al. [51] compared the outcome (objective response to PRRT, PFS, and OS) of two groups of panNET patients depending on whether the primary tumor was resected before PRRT or not. Patients who underwent primary tumor surgery before PRRT showed higher stabilization or objective responses after PRRT (*p* = 0.006) and a better median PFS (70 vs. 30 months; *p* = 0.002) and OS (112 vs. 65 months; *p* = 0.011), as compared to non-operated patients, respectively. The efficacy of PRRT also seems to be affected by a low tumor burden (stage III) and a low proliferation index (G1), associated with a longer PFS [52]. The role of SSA-PET/CT in response to PRRT is still under study. In fact, current ENETS guidelines recommend the use of RECIST criteria to assess response to treatment in panNETs [23]. However, the limitations of a purely morpho-dimensional approach are particularly relevant in the setting of well-differentiated slow-growing tumors. The integration of morphological data with density findings (Choi criteria) to assess treatment response was also implemented in neuroendocrine tumors, and in particular, for panNETs [53,54]. The standard evaluation of response relies on anatomical imaging. However, no definitive response criteria for SSA-PET/CT in NETs were validated, and PET/CT was not routinely performed as interim or end-of-treatment imaging. Many papers tried to establish a correlation of various semiquantitative PET/CT parameters with outcome after PRRT. However, discordant results were reported. Haug et al. used SUVmax and tumor-to-spleen SUV ratio (SUV_T/S_) for early prediction of time to progression (TTP) and for prediction of clinical outcomes after the first PRRT cycle in a cohort of patients with well-differentiated NETs. In particular, the authors calculated the percentage changes (Δ) in SUVmax and SUV_T/S_ relative to the corresponding baseline measurements of up to three tumors in four organs (liver, lung, lymph nodes, and bone), as well as the primary tumor. Any decrease in SUVmax and SUV_T/S_ after the first cycle of therapy was considered a positive response to therapy. Multivariate regression analysis identified ΔSUV_T/S_ as the only independent predictor for tumor progression during follow-up, which also showed a correlation with clinical improvement [55]. Interestingly, ΔSUVmax was not a predictor of TTP. Sharma et al. [56] applied standard PERCIST and modified PERCIST criteria to assess PRRT response with [^68^Ga]Ga-DOTATATE PET/CT. In particular, the authors evaluated baseline SUVmax of a single target lesion (the one with the highest uptake) and of up to five lesions (SUVmax-av), the ratio between tumor SUVmax, and the SUVmean of the spleen (SUV_T/S_) and liver (SUV_T/H_). In patients with baseline and follow-up PET/CT, any change of these parameters was assessed and correlated to PFS. Baseline single lesion SUVmax and SUVmax-av predicted the response to [^177^Lu]Lu-DOTATATE, while only target lesion SUVmax predicted PFS. Neither baseline SUV_T/S_ or SUV_T/H_ were predictive of the response to PRRT. In terms of PERCIST, a change in SUVmax, SUV_T/S_, or SUV_T/H_ did not predict for PFS, while a change in SUVmax-av, according to modified PERCIST, did.

Huizing et al. respectively compared anatomical and receptor imaging criteria, using RECIST 1.1 and Choi for CT imaging and PERCIST criteria for [^68^Ga]Ga-DOTATATE PET/CT, to assess PRRT response. [^68^Ga]Ga-DOTATATE PET/CT proved to be able to detect progression disease earlier than anatomical imaging, but [^68^Ga]Ga-DOTATATE uptake after PRRT was not predictive for OS [57]. Currently, many efforts are being dedicated to establish the best treatment option as first-line treatment. The NETTER-2 trial is in an ongoing phase 3, randomized multicenter trial (NCT03972488) that includes grade 2 and grade 3 advanced GEP-NET patients, comparing the efficacy of the association of [^177^Lu]Lu-DOTATATE with 30 mg octreotide LAR versus high dose (60 mg) octreotide LAR. The ongoing COMPETE trial (NCT03049189) is a phase 3, randomized, multicenter trial that compares the efficacy of PRRT (with [^177^Lu]Lu -DOTATOC) to medical treatment with everolimus as a first-line treatment of advanced GEP-NETs (all grades included). The possibility to establish prognostic parameters in patients affected by an often indolent disease is of the utmost importance. A new approach to image analysis is represented by radiomics. Texture analysis is an emerging tool of diagnostic imaging that may also be used in the setting of prognostic parameters and to assess the response to treatment. A recent multicentric study assessed the prognostic value of intratumoral textural features (TF) determined by the baseline somatostatin receptor (SSTR)-PET/CT in 31 patients scheduled for PRRT: in ROC analysis of TF, entropy demonstrated a significant predictive ability for OS (cutoff = 6.7, AUC = 0.71, *p* = 0.02), with an accuracy of 71%. Increasing entropy could predict longer survival (9 6.7, OS = 2.5 y, 17/31), whereas less entropy portended inferior outcome (G 6.7, OS = 1.9 y) [58]. Önner et al. reported that, among tested radiomic features on pretreatment with [^68^Ga]Ga-DOTA-TATE PET/CT, skewness and kurtosis were able to predict PRRT response [59]. Although radiomics is very appealing, especially since it can provide data in a noninvasive way derived from routinely performed imaging examinations, the results published so far are very preliminary, mainly as a consequence of the lack of standardization of the techniques used for image analysis, segmentation, and interpretation. Further studies are needed to establish the clinical impact of the data that can be derived from texture analysis in the setting of panNETs. Another potentially interesting clinical application is presented by the work of Collamati et al. The authors tried to assess the feasibility of a new radio-guided surgery approach using [^90^Y]Y-DOTATOC in 30 patients with panNETs, after a previous estimation of the tumor uptake by [^68^Ga]Ga-DOTATATE PET/CT, with promising results. This technique, with respect to established *γ*-radio-guided surgery, would allow a clearer delineation of the margins of the pathologic tissue, allowing a more precise tumor resection, together with a substantial reduction of the dose given to the medical staff. In panNETs, surgical excision is the gold standard treatment, and the possibility to minimize the resection of healthy pancreatic parenchyma adjacent to the tumor lesion could be of remarkable importance for the patient’s outcome and quality of life. More specifically, panNET enucleation implies a significantly reduced invasiveness compared to formal pancreatectomy, leading to relevant tissue sparing and less incidence of long-term functional consequences (i.e., post-operative diabetes, exocrine insufficiency) [60].

## 3. [^18^F]FDG

**Figure 2:**
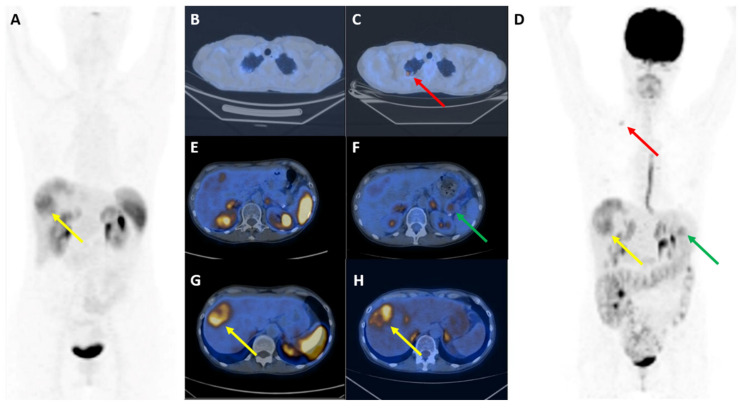
[^68^Ga]Ga-DOTANOC (**A**,**B**,**E**,**G**) and [^18^F]FDG (**C**,**D**,**F**,**H**) images in a G2 panNET patient studied for staging. Discordant pattern of uptake can be documented at lung level (transaxial fused images and MIP (**B**–**D**): red arrows shows FDG-only positivity), at pancreatic primary level (transaxial fused images (**E**,**F**): green arrows show FDG-only positivity), and liver level (**A**,**D**,**G**,**H**: yellow arrows show different distribution of both tracers within the same lesion).

The vast majority of panNETs express somatostatin receptors (SSTR) on cell surfaces, especially in well-differentiated lesions with a low expression of Ki-67 proliferation index (G1 and G2). The SSTR decreases when tumors become differentiated and more aggressive. The loss of SSTR corresponds to the increase of cell glucose utilization [61]. FDG is a glucose analog that remains trapped in the tumor cells proportionally to their glucose metabolic activity, allowing cell visualization of [^18^F]FDG PET/CT images. [^18^F]FDG PET/CT generally shows low sensitivity in small growing well-differentiated neuroendocrine tumors (G1 and G2), while it is of utmost importance in the evaluation and management of high-grade NENs, also providing important prognostic information [62]. Considering that some originally well-differentiated tumors may present dedifferentiation during the disease course, the role of FDG for the assessment of lower grade tumors is an object of active debate. In fact, many papers have investigated the potential clinical role of FDG in NEN; however, the portrayed results were often biased by several factors, including small and heterogeneous patient samples, variable time between pathologic evaluations (used to assess the differentiation grade), and performance of [^18^F]FDG PET/CT, variable primary tumor sites (a factor that is well-known to affect the probability of developing undifferentiated clones during the course of the disease), and tumor grades. Many studies demonstrated a positive correlation between Ki-67 expression and [^18^F]FDG SUVmax [63,64,65]. In patients with Ki-67 < 10%, [^18^F]FDG PET/CT showed a low/absent uptake, while its role was more relevant in patients with higher Ki-67 values [66]. In particular, in a study conducted on 26 pathological-grade panNET patients, [^18^F]FDG PET/CT showed sensitivity of 40% in G1, 60% in G2, and 95% in G3 patients [67]. One of the reasons supporting FDG scanning is the possibility of early identification of undifferentiated clones that affect the patient’s prognosis and outcome. In fact, many reports indicate the high PPV of [^18^F]FDG PET/CT for the detection of potentially aggressive tumors (G2, G3, positive nodes or metastatic spread) [68], suggesting a potential role of [^18^F]FDG PET/CT in panNET prognostication and risk stratification [69]. Recently, Chan et al. [70] proposed a new grading scheme for metastatic NEN derived from data obtained by using dual tracer imaging (SSA-PET/CT and [^18^F]FDG PET/CT): the “NETPET score”. This score identifies five categories of patients, including P1, cases with positive SSA-PET only; P2–P4, intermediate cases with both positive SSA-PET and [^18^F]FDG PET/CT; P5, cases with positive [^18^F]FDG PET/CT only; P0, patients with both negative scans. The NETPET score significantly correlates with tumor grade and overall survival. When positive, [^18^F]FDG PET/CT impacts therapeutical management, indicating the need for a more aggressive treatment regime. The INTERNET study, a multicenter phase II trial, evaluated the efficacy of everolimus in poor prognosis grade 2 (G2) panNETs. [^18^F]FDG PET/CT was performed at baseline during treatment and every 3 months during post-treatment follow-up. Authors found a partial metabolic response on [^18^F]FDG PET/CT in 56% of patients compared to a morphological imaging response rate of 0% as assessed by RECIST alone. The finding of a metabolic response in the absence of a corresponding morphological imaging response suggested that everolimus may reduce the metabolic activity of the tumor without causing apoptosis and that [^18^F]FDG PET/CT scanning can be used as a tool for metabolic treatment response assessment. [^18^F]FDG PET/CT is often required in PRRT protocols and, even though FDG positivity does not exclude the patient from PRRT, several studies have demonstrated that it is certainly useful to predict response. Zhang et al. [71] aimed to determine the role of [^18^F]FDG PET/CT in a large cohort of 495 patients with metastatic NENs who were treated with a long-term follow-up. In the panNEN subgroup (199/495 patients, 40.2%), median OS and PFS were significantly higher in the FDG negative than in the FDG-positive group (median OS: 114.3 vs. 52.8 mo and median PFS: 36.9 vs. 22.4 mo, respectively; for both *p* < 0.001). This was in line with the study of Sansovini et al. [47], conducted only on advanced panNET patients treated with PRRT, in which FDG PET/CT was found to be the only independent prognostic factors for PFS (*p*  =  0.013) in the multivariate analysis. Moreover, more aggressive FDG positive tumors, especially G2 and G3, probably benefit from more intensive therapeutic approaches, such as the combination of PRRT and chemotherapy [72,73]. Current, EANM [24] and ENETS [23,74] guidelines only recommend the use of [^18^F]FDG for the localization of NECs and high-grade poorly-differentiated NETs with aggressive behavior for prognostic stratification and eventually for clarification of equivocal findings on conventional imaging. The ENETS guidelines [22] for well-differentiated panNETs also consider [^18^F]FDG PET/CT to assess tumor burden and prognosis in cases of rapid tumor progression in earlier diagnosed G1–G2 tumors. In clinical practice, the association of these two tracers is often performed, with high regional and national differences, in patients affected by intermediate or high-grade panNETs in different scenarios: at initial diagnosis, when the SSA-PET shows a heterogeneous SSTR expression among different tumor lesions or within the same lesion, in case of a discrepancy between conventional radiological imaging and SSA-PET at the diagnosis or during therapy (earlier identifying the non-responders) and before starting a new line of therapy, such as PRRT [75] (Figure 2). In well-differentiated tumors, G1 and low-grade G2 (Ki-67 < 10%), the role of routine FDG assessment is more controversial: it is certainly agreed that it can provide useful prognostic information if positive; however, it is also expected that most patients will be FDG negative. Therefore, the indication to perform FDG in this setting is generally referred to a multidisciplinary meeting discussion (Table 1). 

## 4. [^18^F]FDOPA

**Figure 3:**
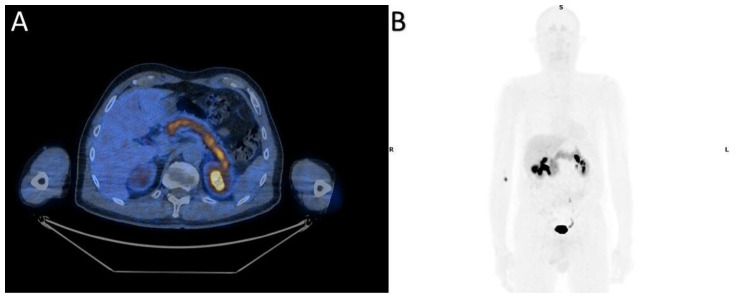
[^18^F]FDOPA PET/CT transaxial images (**A**) and MIP (**B**) show diffuse physiological uptake in the pancreas.

DOPA is an amino acid containing two hydroxyl groups on the third and fourth positions of the phenol ring. It can be labeled with the positron emitter isotope ^18^F in the sixth position, forming [^18^F]FDOPA, which allows PET/CT imaging. [^18^F]FDOPA is a large neutral amino acid that enters the catecholamine metabolic pathway of endogenous l-DOPA. The relatively long half-life (110 min) of [^18^F]FDOPA makes it suitable for transportation to centers without an on-site cyclotron, allowing for more flexible imaging timing, and offers the possibility of acquiring delayed images if needed. The main clinical application of imaging with [^18^F]FDOPA PET/CT is for the assessment of striatum, brain tumors, and NETs, especially from the midgut, as well as congenital hyperinsulinemic hypoglycemia [76]. The disadvantages of the use of DOPA for the assessment of panNET include the lack of information on SRE (that has crucial clinical management relevance), and the reported lower accuracy in lesions detection due to unfavorable biodistribution in the pancreas [77,78] (Figure 3).

Moreover, potential pancreatic false-positive findings in [^18^F]FDOPA PET/CT include various non-neuroendocrine neoplasms, such as solid pseudopapillary pancreatic tumors [79], pancreatic serous adenoma, and granular tissue [80]. Current EANM guidelines, in fact, suggest the use of [^18^F]DOPA for midgut and hindgut NENs, while in foregut tumors, such as panNETs, it is not indicated [24]. The oral pre-administration of carbidopa (100–200 mg 1 h before injection), an inhibitor of DOPA decarboxylase, has been proposed to increase [^18^F]FDOPA uptake by the striatum in brain studies and by tumor cells in the imaging of GEP-NET pheochromocytomas and paragangliomas. In fact, decreased peripheral [^18^F]FDOPA decarboxylation causes a reduced renal clearance of the tracer, thereby increasing tracer availability and uptake by target tissues. Physiologic pancreatic uptake is considerably decreased by carbidopa administration, a finding whose mechanism is still unclear [17]. In the last decade, only one study [81] supported the application of carbidopa-assisted [^18^F]FDOPA PET/CT for 20 non-functioning panNET patients when ^68^Ga-radiolabeled SSAs were not available. A whole-body acquisition (starting between 20 and 30 min after [^18^F]FDOPA injection) was performed in all patients. In selected patients, the [^18^F]FDOPA PET/CT acquisition protocol also included an early acquisition (5 min post-injection) centered over the upper abdomen (one 10 min step). Normal pancreatic parenchyma was only faintly visible in all the included patients, confirming the effective inhibitory influence of carbidopa premedication on the physiologic uptake of the [^18^F]FDOPA. The sensitivity of [^18^F]FDOPA PET/CT for primary panNET detection and for nodal and distant metastatic spread identification (patient-based analysis) was 90%, 81%, and 100%, respectively. There are at least two studies in the literature concluding that [^18^F]FDOPA, combined with carbidopa premedication and an early acquisition centered over the pancreas, is also a valuable diagnostic tool in patients with insulinoma. Leroy-Freschini et al. [82] preoperatively studied 25 patients with insulinoma-related hyperinsulinemic hypoglycemia (HH). In all cases, patient oral carbidopa was administered before PET, and image acquisition consisted of an early scan centered over the pancreas (5 min after [^18^F]FDOPA injection, field of view including the upper abdomen), and a delayed whole-body acquisition, starting 20–30 min later. Using this approach, [^18^F]FDOPA localized insulinoma in 21 of the 25 studies, leading to a primary lesion detection rate of 84%. Four lesions (19%) were only detected on early acquisitions. Similar results were obtained by Imperiale et al. [83], strongly recommending carbidopa premedication associated with early acquisition centered over the pancreas. Carbidopa premedication led to low residual pancreatic [^18^F]FDOPA activity, preserving tumoral uptake with consequent insulinoma detection in more than half of adult patients with HH and more than 70% of patients with a final diagnosis of insulinoma. In a limited series of 10 insulinoma patients, Nakuz et al. [84] reported the clinical usefulness of early acquisition [^18^F]FDOPA PET/CECT, without carbidopa premedication, to detect insulinoma lesions: PET/CT was positive in seven out of 10 patients with histologically verified insulinoma. However, all these studies are hampered by the retrospective design and the small populations. Further studies are necessary to clarify if [^18^F]FDOPA PET/CT, with or without carbidopa premedication, may have a role in insulinoma detection.

## 5. Exendin-4

Insulinomas are the most common functioning endocrine insulin-secreting pancreatic neoplasm, clinically manifesting with often difficult to treat hypoglycemia. Following biological and biochemical confirmation of an insulinoma, preoperative localization is based on computed tomography (CT) or magnetic resonance imaging (MRI) and endoscopic ultrasonography (EUS) [85]. In a low percentage of patients with insulinomas (<5%–10%), all conventional imaging studies are negative due to the small lesion sizes (82% < 2 cm and 47% < 1 cm). Regarding functional imaging, SSA-PET/CT is limited by poor sensitivity for the detection of insulinomas, being positive in approximately 25–31% of cases that present significant SSTR expression [21]. The latest agents being used for the detection of insulinomas are glucagon-like peptide-1 receptor (GLP-1R) agonists, which have been proven to have a high sensitivity compared to SSTR2 analogs [22,86]. GLP-1R is a kind of G protein-coupled receptor which regulates insulin secretion in the pancreatic beta-cells. In the last decade, several GLP-1-like radioligands with high binding affinity to GLP-1R have been developed [86], such as radiolabeled exendin-4, an agonist with strong binding affinity for GLP-1R and resistance to serum degradation [87,88]. PET/CT imaging with [^68^Ga]Ga-DOTA-exendin-4 or [^68^Ga]Ga-NOTA-MAL-Cys39-exendin-4 provides an accurate localization of the primary lesion due to high tumor-to-background ratio, GLP-1 expression levels of insulinoma, and spatial resolution [89,90]. GLP-1 receptor imaging has been reported to be superior to CT, MRI, and SPECT for the detection of small insulinomas. Nevertheless, GLP-1R-based functional imaging has some limitations that may lead to a false negative exam, such as localization of the insulinoma in the pancreatic tail near the left kidney (site of physiological tracer’s excretion), false interpretation of the pancreaticoduodenal region uptake (falsely interpreted as physiological uptake in Brunner glands), or low expression of GLP-1R (especially in the malignant insulinoma subtype) [91,92]. Wild et al. [93], in fact, demonstrated that, in contrast to benign insulinomas, only a low percentage of malignant insulinomas (36%) expressed GLP-1 receptors. Authors carried out a comparison study in 11 patients with malignant insulinoma, performing both GLP-1 receptor imaging (111In-labeled [Lys40(Ahx-DTPA)NH2]-exendin-4 SPECT/CT) and [^68^Ga]Ga-DOTATATE PET/CT. The latter was positive in 73% of malignant insulinomas, and [111In]In-DTPA-exendin-4 SPECT/CT was positive in 36%. Concomitant GLP-1 and SSTR2 expression was discovered in only one patient. Being a whole-body imaging technique, exendin-PET/CT gives important preoperative information on tumor size and localization that is fundamental for surgical planning since resection (enucleation of the lesion or partial pancreatic resection) is the only curative treatment [94]. Brand et al. [95] developed a bimodal imaging probe (PET/fluorescence) for imaging GLP-1R expression in the pancreas and in pancreatic islet cell tumors preoperatively, allowing the diagnosis of primary growths and metastases in a whole-body imaging setting as well as intra-operatively for the real-time detection of tumor margins, infiltrative growth, or residual tumor cells in a surgical cavity. An attempt has also been made to develop tracers based on exendin-4 for PRRT to provide patients with a new line of treatment, especially for those with widespread disease radiolabeled with [^177^Lu] [96]. ^111^In-labeled exendin-4 analogs, predominantly used for imaging of γ-radiation, also emit low energy Auger electrons, which have a tissue penetration of only 0.02–10 μM, exercising their cytotoxic potential when in close proximity to the DNA after internalization. All exendin-4–based tracers show high kidney toxicity, currently limiting the use of PRRT [94]. 

## 6. New Tracers

### 6.1. SSTR Antagonist

Recently, radiolabeled SSTR antagonists have been successfully developed as alternative PET/CT tracers to agonists in patients with well-differentiated panNETs. These tracers are not internalized after receptor binding and show both a high tumor uptake and a long tumor retention (both due to a higher number of binding sites and slower dissociation rates) [97,98]. Several SSTR2 antagonists were developed; however the radiopharmaceutical that encountered wider employment was [^68^Ga]Ga-NODAGA-JR11 (68-Ga-OPS202), a pure SSTR2 ligand, showing the highest affinity for SSTR2 and the best biodistribution profile [99]. Dosimetry studies showed a calculated mean effective injected dose comparable to the agonists and an acceptable radiation dose to organs. The optimal imaging time window was between 1 and 2 h after the injection [99]. It was well tolerated (only three grade 1 adverse events were possibly related to treatment: eosinophilia, rash, and diarrhea). 68-Ga-OPS202 has a renal elimination and a remarkably low accumulation in SSTR2-expressing organs, such as the pituitary gland, adrenals, and the uncinate process of the pancreas, optimizing image reading. Compared to the agonists, one of the most important differences in biodistribution is its lower uptake in normal tissues like the liver, spleen, gastrointestinal tract, and lungs [99]. This higher tumor-to-background ratio can improve tumor detection, as demonstrated in a study conducted on 12 patients with GEP-NETs, in which [^68^Ga]Ga-NODAGA-JR11 (68Ga-OPS202) proved to be more sensitive than [^68^Ga]Ga -DOTATOC (88%–94% vs. 59%), mainly due to a higher detection rate of liver metastasis. The nodal metastasis detection rate was comparable between the two tracers. 68-Ga-OPS202 also had a better reproducibility and positive predictive value [100]. A recent study evaluated the role of another SSTR2 antagonist, [^68^Ga]Ga-DOTA-JR11, in patients with metastatic NETs. This tracer demonstrated a higher detection rate for liver and splenic lesions, lower for bone lesions, and a comparable detection rate for malignant lymph nodes and primary tumors compared to agonists [101]. [^68^Ga]Ga-NODAGA-JR11 (68Ga-OPS202) and [^68^Ga]Ga-DOTA-JR11 differ from the chelator and are directly correlated to the SRRT2 binding affinity [98,102]. The study of Fani et al. [103] in particular indicated that labeling DOTA-JR11 with ^68^Ga reduced its SSTR2 binding affinity by a factor of approximately 80, whereas labeling NODAGA-JR11 with ^68^Ga had no impact on SSTR2 binding. Despite these differences, both antagonists are valid alternatives to SSTR agonists in patients with liver-dominant metastatic NETs [104], thanks to lower liver background activity and corresponding easier detection of even small lesions. Currently, the possible theragnostic role of somatostatin antagonists is under study. Reidy-Lagunes et al. published a phase I trial of well-differentiated NETs treated with radiolabeled somatostatin antagonist [^177^Lu]Lu-satoreotide tetraxetan, including nine patients with panNETs, that proved to deliver high radiation doses to NETs, with favorable tumor-to-normal organ dose ratios. However, the initial treatment schedule of this trial was associated with more severe hematotoxicity than expected from SSTR2 agonists at the same or higher red marrow dose [105].

### 6.2. [^68^Ga]pentixafor

CXCR4 is a member of the chemokine receptor subfamily of seven transmembrane domain G-protein coupled receptors, whose sole known natural ligand is CXCL12/SDF-1, involved in leukocyte recruitment and in fundamental processes, such as the development of the hematopoietic, cardiovascular, and nervous systems during embryogenesis. The receptor has been found to be expressed by multiple cancers, including breast, prostate, lung, colon, and multiple myeloma [106]. Neoplasms with high CXCR4 expression are associated with more aggressive behavior, early metastatic spread, higher risk of relapse, and lower survival. Regarding neuroendocrine tumors, Deschamps et al. found that CXCR4 expression was more common in G2 than in G1 ileal NET and that this expression was associated with a high rate of lymph node metastases and lower survival [107]. The study of Kaemmer et al. supported these findings since they observed an increase in CXCR4 expression from well to poorly differentiated GEP-NENs, a significant correlation with tumor grade between Ki-67 and CXCR4 expression, and a significant negative correlation between CXCR4 expression and overall survival. Patients negative for CXCR4 had increased survival as compared to patients positive for CXCR4 (50.0 vs. 34.0 months; log-rank *p* = 0.068) [108]. Wester et al. developed [^68^Ga]pentixafor (^68^Ga-CPCR4.2), a cyclic pentapeptide that enabled sensitive and high-contrast imaging of human CXCR4 expression in vivo [109]. In a study conducted on 12 patients with GEP-NET, [^68^Ga]pentixafor performance was compared with [^68^Ga]Ga-DOTATOC and [^18^F]FDG, showing that CXCR4 seemed to play only a limited role in detecting well-differentiated NET as there was an inverse expression of SSTR2 and CXCR4 in G1 to G3 NETs, with an elevation in CXCR4 and a decrease in SSTR2 expression with increasing grade. Thus, [^68^Ga]pentixafor PET/CT might serve as a noninvasive tool for evaluating the possibility of CXCR4-directed endoradiotherapy in advanced dedifferentiated SSTR-negative tumors [110].

## 7. Other Tracers

There is limited evidence about the expression of [^68^Ga]Ga-PSMA in neuroendocrine tumors, typically characterized by an increased neovascularization, which also expresses the prostate transmembrane glycoprotein (PSMA). So far, only two cases of [^68^Ga]Ga-PSMA-avid panNET have been described in the literature [111,112]. Other new radiolabeled somatostatin-analogs for (PET) imaging of NEN are currently under study: [^68^Ga]Ga-DATA-TOC, which can potentially be used for the development of an instant kit-type labeling method at room temperature similar to ^99m^Tc-labelled radiopharmaceuticals, increasing the availability of ^68^Ga-labelled somatostatin analogs for routine clinical use [113]; [^18^F]SiTATE, characterized by high tumor uptake, excellent image quality, and a straightforward labeling approach [114]; [^18^F]AlF-NOTA-octreotide ([^18^F]AlF-OC), which has a similar biodistribution to [^68^Ga]Ga-DOTATATE but with lower uptake, especially in the spleen and the salivary glands. [^18^F]AlF-OC has demonstrated good tumor lesion targeting, comparable to [^68^Ga]Ga-DOTATATE, but seems to be more sensitive in liver lesion detection (probably as a result of the lower background uptake in the liver for [^18^F]AlF-OC), and more sensitive in the bone than [^68^Ga]Ga-DOTATATE [115]. However, the mean SUVmax for all lesions is significantly higher for [^68^Ga]Ga-DOTATATE compared with [^18^F]AlF-OC at 1 and 2 h post-injection (*p* = 0.016 and *p* = 0.033), but not at 3 h post-injection (*p* = 0.065).

## 8. Conclusions

The current review offers an overview of all the currently-used radiopharmaceuticals and of new upcoming tracers for panNENs and their clinical impact on therapy management. SSA-PET/CT is indicated as the first-line diagnostic procedure for the staging and restaging of any non-insulinoma panNET case for detection of an unknown primary tumor site or early relapse and for evaluation of the in vivo SRE to select patient candidates for PRRT with ^90^Y or ^177^Lu and/or cold somatostatin analogs. SSA-PET/CT also has a strong impact on clinical management, leading to a change in treatment in approximately one-third of the cases. [^18^F]FDG PET/CT is mostly indicated in patients with high-grade panNENs or with the suspicion of dedifferentiation of G1/G2 panNETs (for example, in cases of rapid progression or mismatch between SSA-PET and conventional imaging) and for prognostic stratification, since FDG positivity is associated with poor prognosis and poor response to PRRT treatment. [^18^F]FDOPA is not indicated for the study of panNETs due to its physiological uptake in the pancreas, and early acquisition of the abdomen and premedication with carbidopa may be useful to increase the accuracy. GLP-1R agonists are particularly useful for benign insulinoma detection, but their accuracy decreases in the case of malignant insulinomas. New upcoming tracers are under study, such as the promising SSTR antagonists, which show a favorable biodistribution and a higher tumor-to-background ratio that increases tumor detection, especially in the liver.

## Figures and Tables

**Table 1:** Summary of the main clinical key points of the two EANM/ENETS recommended radiopharmaceuticals.

Clinical Key Points			
Radiopharmaceuticals	Main Indication	Diagnostic Accuracy	False Positive Findings
[^68^Ga]Ga-DOTATATE, DOTATOC, DOTANOC	staging and restaging any non-insulinoma panNET case; detection of the unknown primary tumour site or early relapse; evaluation in-vivo SRE; selection for PRRT and/or cold SSA	sensitivity: 86 to 100%; specificity from 79 to 100%	pancreatic uncinate process, accessory spleens (including intra-pancreatic, splenules, infectious/inflammatory findings, non-neuroendocrine tumours
[^18^F]FDG	high grade G2, G3 and NEC; prognosis; rapid tumour progression in earlier diagnosed G1–G2 tumours	sensitivity: 40% in G1, 60% in G2; 95% in G3 patients	infectious/inflammatory findings, non-neuroendocrine tumours
